# First case of isolation of *Nocardia wallacei* reported in Mexico

**DOI:** 10.1016/j.nmni.2016.09.009

**Published:** 2016-09-23

**Authors:** J. González-Nava, K. Sánchez-Herrera, N. Ramírez-Durán, H. Sandoval

**Affiliations:** 1) Universidad Autónoma Metropolitana Unidad Xochimilco, Mexico City, Mexico; 2) Facultad de Medicina, Universidad Autónoma del Estado de México, Toluca, Mexico

**Keywords:** Dyspnea, HIV, *Nocardia transvalensis*, *Nocardia wallacei*, nocardiosis, sputum

## Abstract

*Nocardia* species are aerobic, Gram-positive bacteria with branched filaments reported as opportunistic microorganisms associated with infectious diseases of the skin. We report the isolation of *N. wallacei* in Mexico from a 43-year-old man, an HIV-positive construction worker who sought care for difficulty breathing and abundant sputum.

## Introduction

*Nocardia* species are ubiquitous in the environment as saprophytic bacteria. The *Nocardia* genus comprises aerobic, Gram positive and branched bacteria that have the potential to cause localized or disseminated infection [1]. A major risk factor for *Nocardia* infection is being immunocompromised, specifically those with cell-mediated immunity defects, including HIV [2]. Although the incidence and prevalence of the disease are not known, a 2013 study showed that of 8763 cases reported since 1956, 30% (2607 cases) occurred in Mexico, 29% (2555 cases) in Sudan and 16% (1392 cases) in India. However, there are data that strongly suggest that the real disease burden is probably more important than indicated in a meta-analysis by the International Society for Neglected Tropical Diseases (http://www.isntd.org/isntd-disease-briefs/4592435414). The majority of nocardiosis cases are caused by the *N. asteroides* complex and *N. brasiliensis* [3]. This is the first report of isolation of *N. wallacei* in a man with HIV/AIDS and pulmonary failure in Mexico.

## Materials and Methods

### Organism

The strain was obtained from General Hospital “Dr Manuel Gea González” and was identified as *Nocardia* sp. The patient was a 43-year-old man, an HIV-positive construction worker who sought care for difficulty breathing and abundant sputum. The strain was grown at 37°C in brain–heart infusion medium (Bioxon, Becton Dickinson, México) for 7 days, and it was coded as strain Mic-SIDA.

### Phenotype identification

Colony morphology and Gram stain were determinate; 13 tests of acid production from several substrates and antibiotic sensitivity were performed (Table 1). The comparison of the Mic-SIDA strain was made using biochemical tests of *N. wallacei* (ATCC (American Type Culture Collection, Manassas, VA, USA) 49873^T^) against a clinical isolated strain of *N. wallacei* and *N. transvalensis* (ATCC 6865^T^) strain [4].

### Gene sequencing

DNA extractions were performed by the Wizard kit following the manufacturer’s instructions (Promega, Madison, WI, USA). Amplification of the 16S rRNA sequence was carried out using the PCR method. The primers used were 8F: 5′-AGAGTTTGATCMTGGCTCAG-3′ and 1492R: 5′-TACGGYTACCTTGTTACGACTT-3′ [5].

The amplification was performed using the MyTaq DNA Polymerase (Bioline, USA) kit. The reaction parameters were as follows: 30 cycles; predenaturation 5 minutes at 94°C; denaturation 60 seconds at 94°C; annealing 30 seconds at 59°C; extension 60 seconds at 72°C; and finally extension postamplification 10 minutes at 72°C. The products were sequenced in the Macrogen Laboratory (Rockville, MD, USA) and compared with the corresponding GenBank and NZ-Taxon registered sequences [6].

## Results

Colonies were white, with a dusty appearance and irregular borders. The strain was Gram positive, highly branched with large filaments and further fragmentation to rods and coccobacilli. The biochemical analysis indicated similarities with *N. wallacei* type strain but not with *N. transvalensis* species (Table 1).

The 16S rRNA gene sequencing (1354 bp) indicated a 99.17% homology towards *N. wallacei* (ATCC 49873^T^) in GenBank-BLAST (Basic Local Alignment Search Tool; National Center for Biotechnology Information, Bethesda, MD) and 98.66% in NZ-Taxon; but the gene sequence had a 97.94% of similarity to *N. transvalensis* (ATCC 6865^T^) in GenBank (BLAST) and 97.82% in NZ-Taxon databases.

## Discussion

*N. wallacei* species is not well known and may cause rare infections [7], so its identification in a clinical setting can be complicated; however, with joint analysis by biochemical and molecular tests, it is possible identify accurately.

## Conflict of Interest

None declared.

## Figures and Tables

**Table 1:** Comparison of biochemical tests and antibiotic sensitivity between strain Mic-SIDA and reference strains^a^

Test and sensitivity	Mic-SIDA strain	*Nocardia transvalensis* ATCC 6865T	*Nocardia wallacei* ATCC 49873T	*Nocardia wallacei* clinical isolate
Biochemical test				
Arabinose	−	−	−	−
Lactose	−	−	−	−
Mannose	−	−	−	−
d-Sorbitol	−	+	−	−
Trehalose	+	+	+	+
d-Xylose	−	−	−	−
d-Mannitol	−	+	−	−
d-Glucose	+	+	+	+
d-Galactose	+	+	+	+
l-Rhamnose	−	−	−	−
Cellobiose	−	−	−	−
Esculin	+	+	+	+
Casein	−	−	−	−
Antibiotic sensitivity				
Amikacin	R		R	R
Ceftriaxone	S		S	S
Trimethoprim-sulfamethoxazole	S		S/R	S/R

ATCC, American Type Culture Collection (Manassas, VA, USA); R, resistant; S, susceptible.

a Data adapted from Conville *et al*. [4].
